# Autoantibodies to ADAMTS13 in human immunodeficiency virus‐associated thrombotic thrombocytopenic purpura

**DOI:** 10.1111/vox.13738

**Published:** 2024-09-18

**Authors:** Muriel Meiring, Mmakgabu Khemisi, Susan Louw, Palanisamy Krishnan

**Affiliations:** ^1^ Department of Haematology and Cell Biology, Faculty of Health Sciences University of the Free State Bloemfontein South Africa; ^2^ Universitas Business Unit National Health Laboratory Service Bloemfontein South Africa; ^3^ Department of Molecular Medicine and Haematology University of the Witwatersrand Johannesburg South Africa

**Keywords:** ADAMTS13, autoantibodies, HIV, thrombotic thrombocytopenic purpura

## Abstract

**Background and Objectives:**

Thrombotic thrombocytopenic purpura (TTP) is a potentially fatal thrombotic microangiopathic disorder that can result from human immunodeficiency virus (HIV) infection. The pathogenesis involves a deficiency of the von Willebrand factor (vWF) cleaving protease ADAMTS13 (a disintegrin and metalloprotease with thrombospondin motifs member 13) and the presence of anti‐ADAMTS13 autoantibodies. However, there is insufficient information regarding the epitope specificity and reactivity of these autoantibodies. This study aimed to perform epitope‐mapping analysis to provide novel insights into the specific epitopes on ADAMTS13 domains affected by autoantibodies.

**Materials and Methods:**

The study analysed 59 frozen citrate plasma samples from HIV‐associated TTP patients in South Africa, measuring ADAMTS13 activity using Technozyme® ADAMTS13 activity test, total immunoglobulin (Ig) M and IgA antibodies levels using ELISA kit and purifying IgG antibodies using NAb™ Protein G spin columns. A synthetic ADAMTS13 peptide library was used for epitope mapping.

**Results:**

Overall, 90% of samples showed anti‐ADAMTS13 IgG autoantibodies, with 64% of these antibodies being inhibitory, as revealed by mixing studies. Samples with ADAMTS13 antigen levels below 5% showed high anti‐ADAMTS13 IgG autoantibody titres (≥50 IU/mL), whereas those with 5%–10% levels had low autoantibody titres (<50 IU/mL).The metalloprotease, cysteine‐rich and spacer domains were 100% involved in binding anti‐ADAMTS13 IgG antibodies, with 58% of samples containing antibodies binding to the C‐terminal part of the ADAMTS13 disintegrin‐like domain, indicating different pathogenic mechanisms.

**Conclusion:**

The metalloprotease, cysteine‐rich and spacer domains are the primary targets for anti‐ADAMTS13 IgG autoantibodies in patients with HIV‐associated TTP. These findings suggest potential effects on the proteolytic activity of ADAMTS13, highlighting the complex nature of the pathogenic mechanisms involved.

**Highlights**

- The metalloprotease, cysteine‐rich and spacer domains of ADAMTS13 (a disintegrin and metalloprotease with thrombospondin motifs member 13) were constantly (100%) involved in binding anti‐ADAMTS13 immunoglobulin (Ig) G antibodies in human immunodeficiency virus (HIV)‐associated thrombotic thrombocytopenic purpura (TTP).
- All HIV‐associated TTP patients showed IgG autoantibody binding to amino acid residues 645–684 from the spacer domain, suggesting an epitope area with the amino acid sequence ‘QEDADIQVYRRYGEEYGNLTRPDITFTYFQ’ at positions 650–669.
- Anti‐ADAMTS13 IgG antibodies were found in 90% of patients with HIV‐associated TTP (53/59), whereas anti‐ADAMTS13 IgM antibodies were found in 30% of HIV‐associated TTP patients and 64% contained anti‐ADAMTS13 IgA antibodies.

## INTRODUCTION

Thrombotic thrombocytopenic purpura (TTP), a rare but severe haematologic disease, is a member of a closely related group of disorders, thrombotic microangiopathies (TMA). TMA is a group of disease that has microangiopathic haemolytic anaemia and thrombocytopenia due to the formation of microvascular platelet rich thrombi, which causes ischaemic organ dysfunction such as reduction in kidney function and neurological symptoms. TTP is a prevalent systemic TTP that significantly affects the central nervous system and kidneys, although to a lesser extent [1]. Recent investigations reveal that one of the major abnormalities in chronic relapsing TTP is the absence function of a metalloproteinase enzyme, ADAMTS13 (a disintegrin and metalloprotease with thrombospondin motifs member 13). This enzyme controls the generation of specific large forms of von Willebrand factor (vWF) known as ultra‐large vWF multimers (UL‐vWF), which interacts with platelets for haemostasis. Defects or deficiencies (<10%) of ADAMTS13 leads to the accumulation of UL‐vWF multimers in the circulation, eventually forming vWF‐platelet‐rich thrombi under high shear stress conditions manifesting phenotypically as TTP [2, 3, 4, 5]. Autoantibodies to ADAMTS13 can either inhibit or increase the protease's clearance from the circulation, binding to various protease domains, with the cys‐rich/spacer domain being consistently active. A study on patients with acquired TTP found anti‐spacer autoantibodies target three hotspot areas [6, 7, 8].

The characteristically described forms of TTP are rare but a similar condition is now frequently observed in patients infected with the human immunodeficiency virus (HIV) in Sub‐Saharan Africa since the 1980s, the country with the highest HIV infection incidence and also high HIV‐associated with TTP [9, 10]. Recurrent episodes have been identified in HIV‐related TTP at a rate of up to 60% and a mortality rate of between 10% and 30%, which is high, compared with non‐HIV TTP patients. These high mortality rates can be explained by situations such as diagnostic uncontrollable, inability to identify patients at risk and inadequate resources. Hence, diagnostic and prognostic biomarkers need to be established in HIV‐associated TTP [11, 12]. TTP is seen in acquired immunodeficiency syndrome patients with a low helper T cells (CD4+) count (<200 cells/μL) and high viral loads, and the incidence of HIV‐associated TTP was expected to decline with widespread access to anti‐retroviral therapy (ART). However, cases of TTP in HIV infection are still prevalent in South Africa, despite increased access to ART [13, 14, 15]. Recently, TTP is being observed even in HIV infected patients with viral loads below the detectable limit on ART, but the exact primary pathogenesis is not clear [16, 17]. The transmission of HIV‐associated TTP is prospective linked to various mechanisms related to the viral infection. The HIV endothelial cell dysfunction has been considered as important in the pathogenesis of HIV‐associated TTP. Although some studies suggested that endothelial dysfunction may not be the primary cause of TTP, rather that vascular agitation may be the consequence of TTP. The autoimmune dysfunction with autoantibody production and abnormal T‐cell responses may contribute significantly to the reduction of ADAMTS13 in HIV‐associated TTP. HIV infection with a low CD4+ lymphocyte count and a high viral load are associated with an increased incidence of ADAMTS13 autoantibodies [18, 19]. Furthermore, the presence of ADAMTS13 autoantibodies may contribute to severe ADAMTS13 deficiency and trigger HIV‐associated TTP. Several studies have confirmed the importance of autoantibodies to ADAMTS13 in the pathogenesis of HIV‐associated TTP. In some HIV‐associated TTP cases, acquired ADAMTS13 deficiency may occur in the absence of detectable autoantibodies/ autoantibodies that inhibit ADAMTS13 [20]. Even though many reports are discussed in autoantibodies to ADAMTS13 in HIV‐associated TTP as well as in HIV infected people without TTP, but the binding specificity of these autoantibodies however remains unknown. The detection of ADAMTS13 autoantibodies and defining their epitopes on the ADAMTS13 protein in HIV‐associated TTP patients may be of clinical value with disease prognostication and treatment efficacy assessment.

## METHODS AND MATERIALS

### HIV‐associated TTP plasma samples

A study in South Africa examined 59 frozen, anonymized citrate plasma samples from HIV‐associated TTP patients. The samples were collected from across the country and sent to the Specialized Haemostasis laboratory at the University of the Free State. The samples were collected before treatment, and it is stored at −80°C for at least a year for further research. The study received ethical approval from the University of the Free State's Health Science reserach Ethics Committee (UFS‐HSD2019/0027/3007). The study involved National Health Laboratory Service samples, identified by unique laboratory numbers, de‐identified using a double‐blind technique and given random research numbers. The research was conducted with permission from the Free State Department of Health Provincial Research Committee and blood samples from the National Health Laboratory Service (FS‐201903‐005).

### Mixing study

The study aimed to identify neutralizing immunoglobulin (Ig) G autoantibodies to ADAMTS13 in HIV‐associated TTP plasma samples with ADAMTS13 activity levels below 10%. The Technozyme® ADAMTS13 activity test was used to measure ADAMTS13 activity in samples and pooled normal plasma (PNP). The results showed normal ADAMTS13 activity levels in PNP, ranging from 50% to 150%. However, no correction was shown in the mixing test, indicating the presence of an inhibitor, resulting in less than 50% ADAMTS13 activity.

The Bethesda technique was used to measure the potency of neutralizing anti‐ADAMTS13 antibodies. A Bethesda unit (BU) is the concentration of an inhibitor in plasma that reduces ADAMTS13 activity by 50% in PNP. Residual activity of 25%–75% indicates an inhibitor, whereas over 75% activity indicates the absence of a clinically significant inhibitor. The strength of neutralizing anti‐ADAMTS13 antibodies was evaluated using a modified Bethesda test [21].

The study utilized HIV‐associated TTP plasma samples treated at 56°C for an hour to remove endogenous ADAMTS13 activity. Samples with strong inhibitors were diluted with saline and PNP and incubated at 25°C for 2 h; PNP served as a negative control. ADAMTS13 activity was measured using the Technozyme® ADAMTS13 activity assay.

### Total IgM and IgA antibodies

The study examined IgM and IgA antibodies in samples with a positive titre of anti‐ADAMTS13 IgG antibodies. Total plasma Ig levels were measured using a commercially available ELISA kit from Bethyl Laboratories. The test was conducted in two separate studies using ELISA 96‐well plates pre‐coated with either IgM or IgA anti‐human antibodies. The standard and samples was diluted using dilution buffer according to the manufacturer's instructions. The study involved duplicate wells with standard and sample solutions, incubating them at room temperature for an hour, washing them four times with wash buffer, adding anti‐human IgM/IgA detection antibody, incubating for 1 h, adding horseradish peroxidase (HRP) solution and adding tetramethylbenzidine substrate solution. The reaction was stopped with adding stop solution, and the absorbance was measured at 450 nm using a microplate reader. The manufacturer estimated IgM and IgA concentrations in samples using an extrapolated standard curve, with representative reference ranges for healthy individuals for IgA is 1.1–2.6 mg/mL and for IgM is 0.23–1.4 mg/mL in sodium citrate plasma [6].

### Extraction of IgG autoantibodies

The study purified total IgG antibodies from 53 HIV‐associated TTP plasma samples using NAb™ Protein G spin columns. Positive anti‐ADAMTS13 IgG antibody titres were used for analysis. Protein content and purity of eluted IgG fractions were measured using a BioDrop spectrophotometer. Fractions with an absorbance ratio of less than 0.6 showed no nucleic acid contamination. The purified antibodies were dialyzed in phosphate buffered saline (PBS) to eliminate low molecular weight compounds and salt contamination. The eluted pure IgG protein was pooled and stored at 4°C. The absorbance of the samples were measured at 260/280.

### Epitope mapping studies of anti‐ADAMTS13 IgG antibodies

#### Synthetic peptides

**FIGURE 1:**
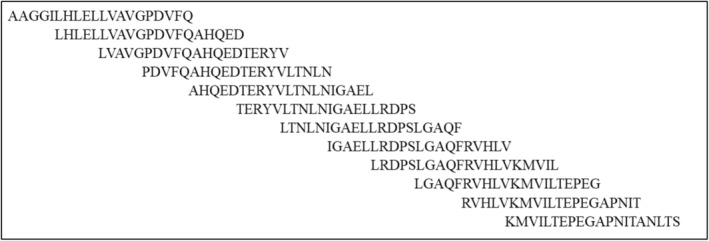
Overlapping linear peptide sequences from the metalloprotease domain 75–150. The designed peptides are 20 amino acids long with 15 overlapping amino acids and an offset of 5 amino acids.

GenScript™ has developed a synthetic peptide library from the ADAMTS13 protein, containing 105 biotinylated peptides. The library was screened for linear B‐cell epitopes using an ELISA‐based method. The peptide library includes domains influencing ADAMTS13 function and vWF binding under static conditions. The N terminus of the synthesized peptides was biotinylated. To minimize costs, the library included domains that significantly contribute to ADAMTS13 function and vWF binding under static conditions. The peptides were designed in a 20‐mer/15‐mer overlapping format with an offset of 5 amino acids (Figure 1). The peptides were extracted as a lyophilized powder with over 75% purity and stored at −20°C. Peptide names were derived from domain names, and the amino acid locations were compared to the full‐length ADAMTS13 protein coding region (Table 1).

**TABLE 1:** The ADAMTS13 domain groupings selected for designing a peptide library.

Domain grouping	Position in ADAMTS13 amino acid sequence	Structure–function
Metalloprotease–disintegrin domains	75–383	Catalytic domains.
Cysteine‐rich spacer domains	440–680	Critical role in substrate recognition and binding, promoting proteolysis of vWF by ADAMTS13

Abbreviations: ADAMTS, a disintegrin and metalloprotease with thrombospondin motifs member 13; vWF, von Willebrand factor.

#### Developing a peptide ELISA

A peptide ELISA was developed using artificial peptides from a library and a monoclonal antibody specific to the Fc‐region of the anti‐human IgG antibody. Overall, 53 HIV‐associated TTP plasma samples were used to generate purified ADAMTS13 IgG autoantibodies, which were used to identify potential epitope sites.

#### Peptide ELISA

Epitope mapping studies were conducted on 105 overlapping biotinylated peptides in a peptide library to monitor binding events in each patient's purified IgG sample using Peptide ELISA, with all synthetic peptides tested individually, with a purity of over 75%.

A 96‐well ELISA plate first pre‐coated with 100 μL/well of streptavidin (200 ng/mL, GenScript®, USA) was diluted in PBS buffer and incubated overnight at 4°C, and the plates were washed four times using washing buffer (PBS/0.05% Tween‐20 [pH 7.4]). After washing, the plates were blocked with 200 μL/well of blocking buffer for 2 h at 37°C. After incubation, the plates were washed again and 100 μL/well of each diluted peptide was added to the precoated plate and incubated at 37°C for 2 h. After incubation, the plates were washed. Then the plates were blocked again with 200 μL/well of blocking buffer for 2 h at 37°C. Following another washing step, 100 μL/well of the purified IgG antibody from each patient was diluted in duplicate to each plate. The plates were incubated for 1 h at 37°C and then washed again. Then, 100 μL/well of an HRP‐conjugated monoclonal anti‐human IgG antibody (Abcam®) was added for detection. The plates were incubated for 1 h at room temperature, followed by another wash. Finally, 100 μL/well of the O‐phenylenediamine dihydrochloride substrate was added and incubated for 10 min at room temperature; thereafter 30 μL/well stop solution (4 M H_2_SO_4_) was added to stop the reaction, and the absorbance was measured at 490–630 nm.

The study involved subtracting the mean optical density at 490 nm (OD_490_) value of two blanks from all other OD_490_ values, with each plate having a negative control. Samples with OD_490_ values greater than the cut‐off value were considered positive binding to the peptide, whereas those with OD_490_ values less than the cut‐off value were considered no binding to the respective peptide.

#### Data analysis

The data were assessed using GraphPad Prism software version 6. Statistical analysis involved the application of a student‐*T* test, and a *p*‐value of <0.05 was deemed to indicate statistical significance.

## RESULTS

### Laboratory inclusion criteria for HIV‐associated TTP diagnosis

The Table 2 shows that the diagnostic criteria for HIV‐associated TTP include laboratory findings of HIV infection, thrombocytopenia, microangiopathic haemolytic anaemia and elevated serum lactate dehydrogenase levels. A creatinine test was also used to assess renal function, with renal impairment considered when creatinine levels were above 90.0 μg/L.

**TABLE 2:** Laboratory inclusion criteria for HIV‐associated TTP diagnosis.

Laboratory test (units)	Laboratory findings	
	Normal values/ranges	HIV–TTP patients
Full blood count		
Schistocytes on morphological examination of slide	Absent	Present
Platelet count (10^9^/L)	150–450	<100
Haemoglobin (g/dL)	12.1–17.2	<12.1
Lactate dehydrogenase (LDH) (U/L)	100–190	Elevated and frequently >800
Creatinine (μg/L)	49–90	>90
Coombs test	Negative	Negative
ADAMTS13 antigen levels (%)	50–150	Usually <5
ADAMTS13 activity (%)	50–150	Usually <10
ADAMTS13 autoantibodies	Negative	Positive
HIV status	Negative	Positive
Anti‐retroviral therapy (ART) at presentation	N/A	Either ART naïve or on ART therapy
Helper T cells (CD4+ count, cells/mm^3^)	500–1500	219.372

Abbreviations: ADAMTS, a disintegrin and metalloprotease with thrombospondin motifs member 13; HIV, human immunodeficiency virus; TTP, thrombotic thrombocytopenic purpura.

### Anti‐ADAMTS13 IgG antibody concentration and Bethesda inhibitory (BU) activity were measured in HIV‐associated TTP plasma samples

Mixing tests were conducted on HIV‐associated TTP patient samples with ADAMTS13 activity below 10% and positive anti‐ADAMTS13 IgG antibody titre. Overall, 53 samples showed inhibitory anti‐ADAMTS13 IgG antibodies, with non‐inhibitory 17, low inhibition 17 and high inhibition 19, respectively. Table 3 shows concentrations and Bethesda Units (BU) for non‐inhibitory, mild inhibitory and high inhibitory of autoantibody titres.

**TABLE 3:** The concentration of anti‐ADAMTS13 IgG antibody in HIV‐associated TTP plasma samples.

Anti‐ADAMTS13 IgG antibodies	Number of samples	Median anti‐ADAMTS13 IgG antibody titre (μg/mL)	Median Bethesda unit (BU/mL)
Non‐inhibitory	17/53	26 (17–223)	<0.5
Low inhibition <5 BU	17/53	42 (18–86)	1.85 (0.64–4.54)
Strong inhibition >5 BU	19/53	96 (32–175)	9.74 (5.10–17.92)

*Note*: Results are expressed as mean ± SD, *p* < 0.05; median anti‐ADAMTS13 IgG antibodies concentration units are expressed as μg/mL and median Bethesda unit is expressed as BU/mL.

Abbreviations: ADAMTS, a disintegrin and metalloprotease with thrombospondin motifs member 13; HIV, human immunodeficiency virus; Ig, immunoglobulin; TTP, thrombotic thrombocytopenic purpura.

### Determination of total IgM and IgA in HIV‐associated TTP plasma samples

Table 4 shows the total IgM and IgA antibody concentrations in HIV‐associated TTP patient (53) samples.

**TABLE 4:** Determination of total IgM and IgA in HIV‐associated TTP plasma samples.

Parameter	HIV‐associated TTP (*n* = 53)
Median IgM level (ranges) Mean ± SD	1.6 (1.05–2.35) mg/mL 1.59 ± 0.27
Median IgA level (ranges) Mean ± SD	1.85 (1.06–2.98) mg/mL 2.10 ± 0.60

*Note*: Results are expressed as mean ± SD, *p* < 0.05, IgM and IgA antibodies concentration expressed as mg/mL.

Abbreviations: HIV, human immunodeficiency virus; Ig, immunoglobulin; *n*, number of samples; TTP, thrombotic thrombocytopenic purpura.

### Determination of anti‐ADAMTS13 IgM and IgA antibodies

**FIGURE 2:**
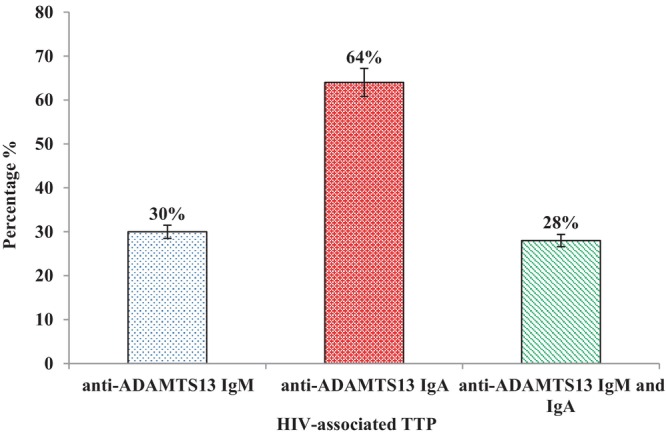
Percentage of human immunodeficiency virus (HIV)‐associated thrombotic thrombocytopenic purpura (TTP) patients with positive anti‐ADAMTS13 (a disintegrin and metalloprotease with thrombospondin motifs member 13) immunoglobulin (Ig) M, IgA and both IgM and IgA autoantibody levels: Results are expressed as percentage (%).

Figure 2 shows the percentage of patients with anti‐ADAMTS13 IgM and IgA antibodies in HIV‐associated TTP plasma samples.

### Binding of purified IgG antibodies to linear overlapping ADAMTS13 peptides using peptide ELISA

Table 5 reveals that 94% of HIV‐associated TTP patient plasma samples have IgG autoantibodies that bind to linear peptides from all four ADAMTS13 proximal domains. In total, 42% of samples have IgG autoantibodies bound to the metalloprotease domain, whereas 58% are bound to both metalloprotease and disintegrin‐like domains. None respond solely to the disintegrin‐like domain, but 98% respond to cysteine‐rich and spacer domains.

**TABLE 5:** ADAMTS13 domains with reactivity towards IgG antibodies of individual HIV‐associated TTP plasma samples.

ADAMTS13 domains	HIV‐associated TTP plasma (*n* = 53)
Metalloprotease domain only	22/53 (42%)
Disintegrin‐like domain only	0/53 (0%)
Both metalloprotease and disintegrin‐like domains together	31/53 (58%)
Cysteine‐rich domain only	0/53 (0%)
Spacer domain only	1/53 (2%)
Both cistein‐rich and spacer domains together	52/53 (98%)
All four ADAMTS13 domains together	31/53 (78%)

*Note*: All results units are expressed as percentages (%).

Abbreviations: ADAMTS, a disintegrin and metalloprotease with thrombospondin motifs member 13; HIV, human immunodeficiency virus; Ig, immunoglobulin; *n*, number of samples; TTP, thrombotic thrombocytopenic purpura.

**FIGURE 3:**
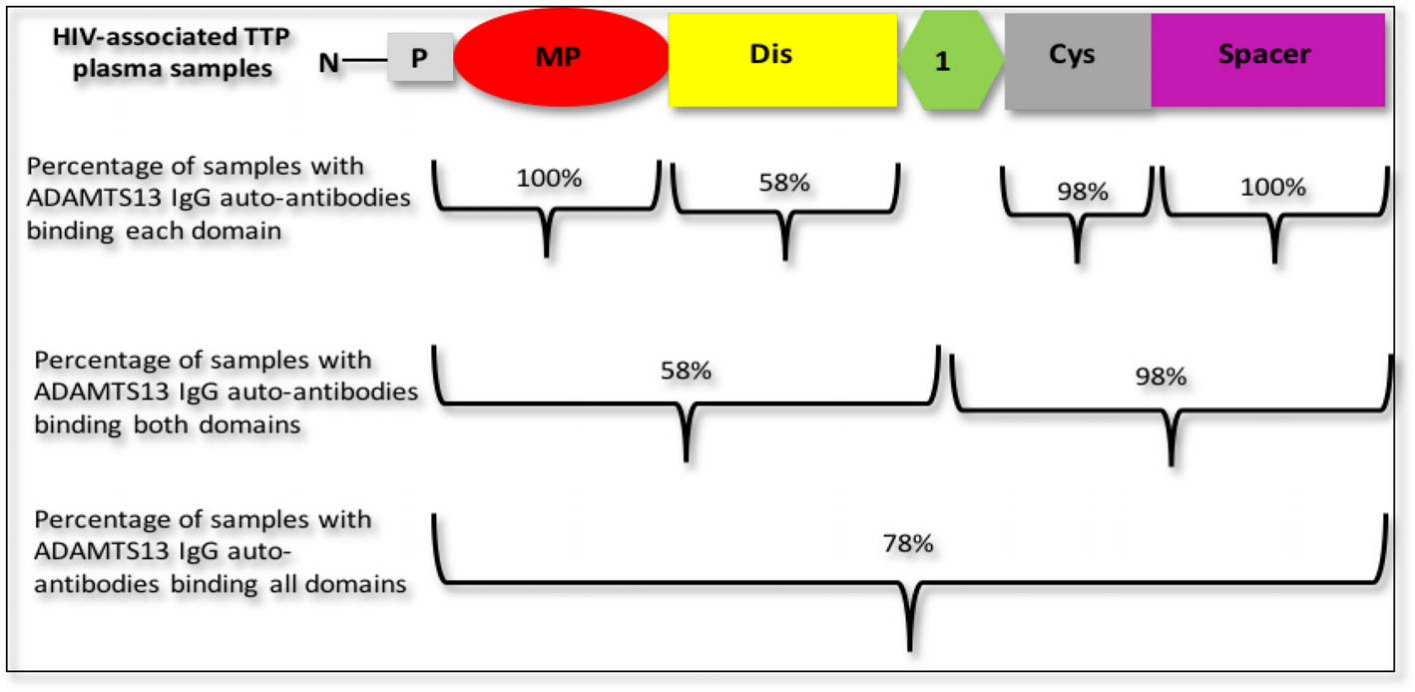
Percentage of human immunodeficiency virus (HIV)‐associated thrombotic thrombocytopenic purpura (TTP) samples with ADAMTS13 (a disintegrin and metalloprotease with thrombospondin motifs member 13) domain‐specific autoantibodies: Cys, cysteine‐rich domain; Dis, disintegrin‐like domain; MP, metalloprotease domain; N, N‐terminal side; P, propeptide; Spacer, Spacer domain; TSP1, thrombospondin motif 1.

Figure 3 shows the percentage of HIV‐associated TTP samples with ADAMTS domain‐specific autoantibodies, with ADAMTS13 metalloprotease, disintegrin‐likes, cysteine‐rich and spacer domains as predominant antibody binding targets. Table 6 summarizes immunoglobulin IgG autoantibody binding data and linear ADAMTS13 peptide epitopes.

**TABLE 6:** Shared and non‐shared linear ADAMTS13 peptide epitope regions that bind to IgG autoantibodies isolated from HIV‐associated TTP patients.

Binding domains	Metalloprotease domain aa epitope regions	Disintegrin‐link domain aa epitope regions	Cysteine‐rich domain aa epitope regions	Spacer domain amino aa regions
Shared epitope regions	75–80	169–189	445–479	595–619
	125–139	320–345	455–469	625–649
	200–224	350–379	475–504	650–669
	260–284	340–379		
Non‐shared epitope regions	80–124	275–304	505–529	560–586
	220–244	290–324	540–564	
	240–26	355–376		
		365–374		

Abbreviations: aa, amino acid; ADAMTS, a disintegrin and metalloprotease with thrombospondin motifs member 13; HIV, human immunodeficiency virus; Ig, immunoglobulin; *n*, number of samples; TTP, thrombotic thrombocytopenic purpura.

Table 7 shows potential antigenic regions in metalloprotease and disintegrin‐like domains, while Table 8 shows potential antigenic regions in cysteine‐rich and spacer domains.

**TABLE 7:** Linear peptides with potential antigenic regions in the metalloprotease (MP) and disintegrin (Dis)‐like domains.

Peptide name	Peptide sequence	Position	HIV‐associated TTP patient group (*n* = 53)
MP1	AAGGILHLELLVAVGPDVFQ	75–94	53/53 (100%)
MP1–MP8	LHLELLVAVGPDVFQAHQEDTERYVLTNLNIGAELLR DPSLGAQFRVHLV	80–129	0/53
MP9–MP10	LRDPSLGAQFRVHLVKMVILTEPEG	115–139	53/53 (100%)
MP9–MP12	LRDPSLGAQFRVHLVKMVILTEPEGAPNITANLTS	125–149	0/53
MP18–MP21	TINPEDDTDPGHADLVLYITRFDLELPDGNRQVRG	160–194	15/53 (28%)
MP19–MP20	DDTDPGHADLVLYITRFDLELPDGN	165–189	37/53 (70%)
MP19–MP21	DDTDPGHADLVLYITRFDLELPDGNRQVRG	165–194	4/53 (8%)
MP25–MP28	VTQLGGACSPTWSCLITEDTGFDLGGVTIAHEIGHS	195–229	10/53 (19%)
MP29–MP34	GFDLGVTIAHEIGHSFGLEHDGAPGSGCGPSGHVMASDGAAPRAG	215–249	0/53
MP33–MP36	DGAPGSGCGPSGHVMASDGAAPRAGLAWSPCSRRQ	235–269	3/53 (6%)
MP37–MP39	APRAGLAWSPCSRRQLLSLLSAGRARCVWD	255–284	26/53 (49%)
MP37–MP/Dis40	APRAGLAWSPCSRRQLLSLLSAGRARCVWDPPRPQ	255–289	25/53 (47%)
MP40–MP/Dis44	LLSLLSAGRARCVWDPPRPQPGSAGHPPDAQPGLY YSANE	270–304	0/53
MP/Dis41–Dis44	SAGRARCVWDPPRPQPGSAGHPPDAQPGLYYSANE	275–309	0/53
MP/Dis43–Dis46	PPRPQPGSAGHPPDAQPGLYYSANEQCRVAFGPKA	285–319	1/53 (2%)
Dis44–Dis47	PGSAGHPPDAQPGLYYSANEQCRVAFGPKAVACTF	290–324	2/53 (4%)
Dis45–Dis48	HPPDAQPGLYYSANEQCRVAFGPKAVACTFAREHL	295–334	1/53 (2%)
Dis49–Dis52	FGPKAVACTFAREHLDMCQALSCHTDPLDQSSCSR	315–349	4/53 (7%)
Dis49–Dis/TSP1 59	FGPKAVACTFAREHLDMCQALSCHTDPLDQSSCSR LLVPLDGTECCGVEKWCSKGRCRSLVELTPIAAVH	315–383	5/53 (9%)
Dis53–Dis58	LSCHTDPLDQSSCSRLLVPLLDGTECCGVEKWCSKGRCRSLVELTP	335–379	5/53 (9%)
Dis53–Dis/TSP1 59	LSCHTDPLDQSSCSRLLVPLDGTECCGVEKWCSKGRCRSLVELTPIAAVH	335–383	5/53 (9%)
Dis55–Dis/TSP1 59	SSCSRLLVPLLDGTECGVEKWCSKGRCRSLVELTP IAAVH	345–383	2/53 (4%)
Dis56–Dis/TSP1 59	LLVPLLDGTECGVEKWCSKGRCRSLVELTPIAAVH	350–383	2/53 (4%)
Dis57–Dis/TSP1 59	LDGTECGVEKWCSKGRCRSLVELTPIAAVH	355–383	1/53 (2%)
Dis58–Dis/TSP1 59	CGVEKWCSKGRCRSLVELTPIAAVH	360–383	253 (4%)
Dis/TSP1 59	WCSKGRCRSLVELTPIAAVH	365–33	11/53 (21%)

*Note*: Amino acid residues in the overlapping regions of antigenic peptides in red. Peptide names are derived from the relevant domain names and amino acid position relative to the coding region of full‐length ADAMTS13 protein.

Abbreviations: ADAMTS, a disintegrin and metalloprotease with thrombospondin motifs member 13; HIV, human immunodeficiency virus; *n*, number of samples; TTP, thrombotic thrombocytopenic purpura.

**TABLE 8:** Linear peptides with potential antigenic regions detected from the Cysteine‐rich (Cys) and Spacer (Spa) domains.

Peptide name	Peptide sequence	Position	HIV‐associated TTP group *n* = 53
Cys1–Cys4	KTQLEFMSQQCARTDGQPLRSSPGGASFYHWGAAV	440–474	2/53 (4%)
Cys3–Cys6	CARTDGQPLRSSPGGASFYHWGAAVPHSQGGDALCR	450–484	8/53 (15%)
Cys2–Cys5	FMSQQCARTDGQPLRSSPGGASFYHWGAAVPHSQG	445–479	23/53 (43%)
Cys3–Cys5	CARTDGQPLRSSPGGASFYHWGAAVPHSQG	450–479	6/53 (11%)
Cys3–Cys10	CARTDGQPLRSSPGGASFYHWGAAVPHSQGDALCR HMCRAIGESFIMKRGHMCRAIGESFIMKRGDSFLD	450–504	4/53 (8%)
Cys7–Cys10	WGAAVPHSQGDALCRHMCRAIGESFDALCRHMCR AIGESFIMKRGDSFLD	470–504	11/53 (21%)
Cys13–Cys16	DSFLDGTRCMPSGPREDGTLSLCVSGSCRTFGCDG	500–534	7/53 (13%)
Cys17–Cys/Spa20	SLCVSGSCRTFGCDGRMDSQQVWDRFCQVCGGGDNST	520–554	29/53 (55%)
Cys18–Cys/Spa20	GSCRTFGCDGRMDSQQVWDRCQVCGGDNST	525–554	1/53 (2%)
Cys13–Cys/Spa20	DSFLDGTRCMPSGPREDGTLSLCVSGSCRTFGCDG RMDSQQVWDRCQVCGGDNST	500–554	5/53 (9%)
Spa24–Spa 28	CSPRKGSFTAGRAREYVTFLTVTPNLTSVYIANHRPLFTH	555–594	1/53 (2%)
Spa31–Spa 34	PLFTHLAVRIGGRYVVAGKMSISPNTTYPSLLEDG	590–624	40/53 (75%)
Spa31–Spa39	PLFTHLAVRIGGRYVVAGKMSISPNTTYPSLLEDGRVEYR VALTEDRLPRLEEIRIWGPL	590–649	2/53 (4%)
Spa37–Spa40	LLEDGRVEYRVALTEDRLPRLEEIRIWGPLQEDAD	620–654	27/53 (51%)
Spa42–Spa/TSP2 46	IWGPLQEDADIQVYRRYGEEYGNLTRPDITFTYFQPKPRQ	645–684	53/53 (100%)

*Note*: Amino acid residues in the overlapping regions of antigenic peptides are highlighted in red. Peptide names are derived from the relevant domain names and the relevant amino acid position relative to the coding region of full‐length ADAMTS13 protein.

Abbreviations: ADAMTS, a disintegrin and metalloprotease with thrombospondin motifs member 13; HIV, human immunodeficiency virus; *n*, number of samples; TTP, thrombotic thrombocytopenic purpura.

## DISCUSSION

TTP is a prevalent type of TTP in Sub‐Saharan Africa; it is primarily caused by HIV infection. The ADAMTS13 protein plays a central role in the pathogenesis of acquired TTP, with autoantibodies targeting this enzyme often being the primary cause of severe ADAMTS13 deficiency in HIV‐associated TTP [9]. Several studies have underscored the importance of measuring autoantibodies to ADAMTS13 in managing patients with TTP. However, the ADAMTS13 autoantibody status in HIV‐associated TTP patients has not yet been fully investigated [20, 22].

The laboratory inclusion criteria used for this diagnosis are summarized from multiple published studies [23, 24]. TTP is a common condition in patients with advanced HIV disease the severe deficiency of ADAMTS13, lower platelet, haemoglobin, CD4+ T‐cell count and high level of plasma lactate dehydrogenase (LDH) and creatinine levels [25, 26]. This study also find similar representing severe deficiency of ADAMTS13 antigen and activity levels, lower platelet count, decreased haemoglobin, low CD4+ T‐cell counts and elevated LDH and creatinine levels in HIV‐associated TTP patients.

The frequency of autoantibodies in acquired TTP patients suggesting that an immune‐mediated activity against ADAMTS13 is present in almost all patients presenting with HIV‐associated TTP [22, 27]. Increased anti‐ADAMTS13 IgG antibody titres are associated with poor prognosis in TTP patients [20]. In concurrence with the previous reports, in the current investigation, the anti‐ADAMTS13 IgG concentration was assessed in 90% of the positive anti‐ADAMTS13 IgG antibodies in this HIV‐associated TTP (53/59) plasma, this indicates the immune‐mediated activity against HIV‐associated TTP patients.

This study also investigated the laboratory evidence of autoimmunity in HIV‐associated TTP plasma samples by also measuring IgM and IgA titres to emphasize the relationship between the disease and the immunological parameters. A relationship between autoantibodies and CD4+ T‐lymphocyte count has previously been documented [19, 23, 24]. It is reported that autoantibodies can trigger T‐cell apoptosis by crosslinking Ig‐related T‐cell membrane molecules and envelope glycoprotein, a glycoprotein on the HIV envelope, resulting in CD4+ T‐cell reduction with loss of integrity of the immune system [25, 26]. Emerging data demonstrate that the HIV‐associated TTP plasma samples were detected with slightly increased plasma IgM and IgA antibodies and had a CD4+ count of less than normal ranges. HIV infection therefore prompts patients to autoimmune responses. Autoantibodies have prognostic significance in infectious diseases such as infections with HIV and have diagnostic value in HIV‐associated TTP.

The IgG antibodies are primarily involved in the pathogenesis of other acquired forms of TTP by causing decreased ADAMTS13 protein in plasma, but these antibodies have not been characterized in acquired HIV‐associated TTP. The selected ADAMTS13 proximal domains include the metalloprotease, disintegrin‐like, cysteine‐rich and spacer domains interact with unravelled vWF substrate and are necessary for the proteolytic activity of ADAMTS13. Furthermore, their activity towards vWF fragments under static conditions has been evaluated and representing the specific effects on ADAMTS13 activity [6, 27]. The results of the current study show that all IgG antibodies isolated from 53 HIV‐associated TTP plasma samples had multiple binding sites on the four functional domains of ADAMTS13 probed. Our epitope‐mapping studies indicated that anti‐ADAMTS13 IgG antibodies identified similar immuno‐dominant epitopes in the HIV‐associated TTP group but additional binding sites were also identified in this group. We also observed that the cysteine‐rich and spacer domains strong major binding sites in the HIV‐associate TTP patients.

The propeptide metalloprotease domain is reported to function as a molecular safeguard of ADAMTS13 and does not affect the enzymatic action of the protein or its expression levels [28], and it also detected IgG antibodies binding the propeptide domain in 20% of acquired TTP plasma samples [7]. This study also identified the first immune‐dominant epitope regions in the metalloprotease domain. All the HIV‐associated TTP samples showed reactivity to the first peptide of the metalloprotease domain. The potential epitope region was identified and comprised of amino acids ‘AAGGI’ at position 75–80 in the full ADAMTS13 nucleotide sequence. These amino acid residues are located on the C‐terminal part of the propeptide domain on the ADAMTS13 protein.

The metalloprotease domain regions contain the ADAMTS13 catalytic sequence as well as ADAMTS13 sub‐sites, which are important for ADAMTS13 interaction with vWF and disintegrin‐like domain has been reported to significantly increase the cleavage efficiency and specificity of ADAMTS13 [29]. In concurrence with the previous reports, we observed the disintegrin‐like domain contains exocites at Arg349 and Leu350 residues that form weaker interactions with the unravelled vWF A2 domain residues at Asp1614 and Ala1612 close to the cleavage site. Thus, antibodies that bind to both the metalloprotease domain and the disintegrin‐like domain may affect the ability of the ADAMTS13 protease to interact with the vWF substrate. Furthermore, the metalloprotease domain was identified as the most antigenic region when compared to the disintegrin‐like domain in HIV‐associated TTP group.

The cysteine‐rich and spacer domains are constantly involved in antibody binding in patients with acquired TTP [7, 8, 30, 31]. The cysteine‐rich and spacer domain have been found valuable for efficient in vivo vWF ADAMTS13 proteolysis. The spacer domain is essential for proteolysis of full‐length vWF under flow conditions [29, 32, 33], and data in the present study also agree with the previous studies also found that 98% of HIV‐associated TTP patient samples had IgG autoantibodies that bind to both the cysteine‐rich as well as the spacer domains. This IgG autoantibodies that bind to these domains may interfere with ADAMTS13–vWF interaction.

Limitation of this study includes the overlapping peptide library derived from the ADAMTS13 protein, synthesized by GenScript (USA), consisting of 105 biotinylated peptides. These peptides were designed to identify linear B‐cell epitopes using an ELISA‐based method. Due to the high costs of peptide libraries for large proteins, only specific domains of ADAMTS13—the metalloprotease, disintegrin‐like, cysteine‐rich and spacer domains were selected, based on their functional significance and binding to vWF under static conditions.

In conclusion, this study showed that the ADAMTS13 proximal domains contain various epitope regions for anti‐ADAMTS13 IgG autoantibody interaction in HIV‐associated TTP patients. Therefore, it is evident that a polyclonal mixture of anti‐ADAMTS13 IgG antibodies is present in HIV‐associated TTP patients with similar binding patterns interacting with specific epitopes in the ADAMTS13 proximal domains. These include amino acid residues 125–139, 169–189, 200–224, 220–244 and 260–284 in the metalloprotease domain, 445–504 and 505–564 in the cysteine‐rich domain and 650–669, 590–624 and 625–649 in the spacer domain. This study has improved our understanding of the immunological response potentially involved in HIV‐associated TTP. This study also recommends screening for inhibitory anti‐ADAMTS13 IgG autoantibodies to characterize the pathophysiology of HIV‐associated TTP.

## CONFLICT OF INTEREST STATEMENT

The authors declare no conflicts of interest.

## Data Availability

The data for this manuscript are available upon reasonable request and is subject to ethics committee approval.
